# Supplementary figures and images for: First isolation of viable Toxoplasma gondii from a black mangabey (Lophocebus aterrimus) reveals the emergence of the Africa 1 lineage in East Asia
Source: PLoS Negl Trop Dis. 2025 Jul 23;19(7):e0013133. doi: 10.1371/journal.pntd.0013133 (PMC12286360; doi:10.1371/journal.pntd.0013133)

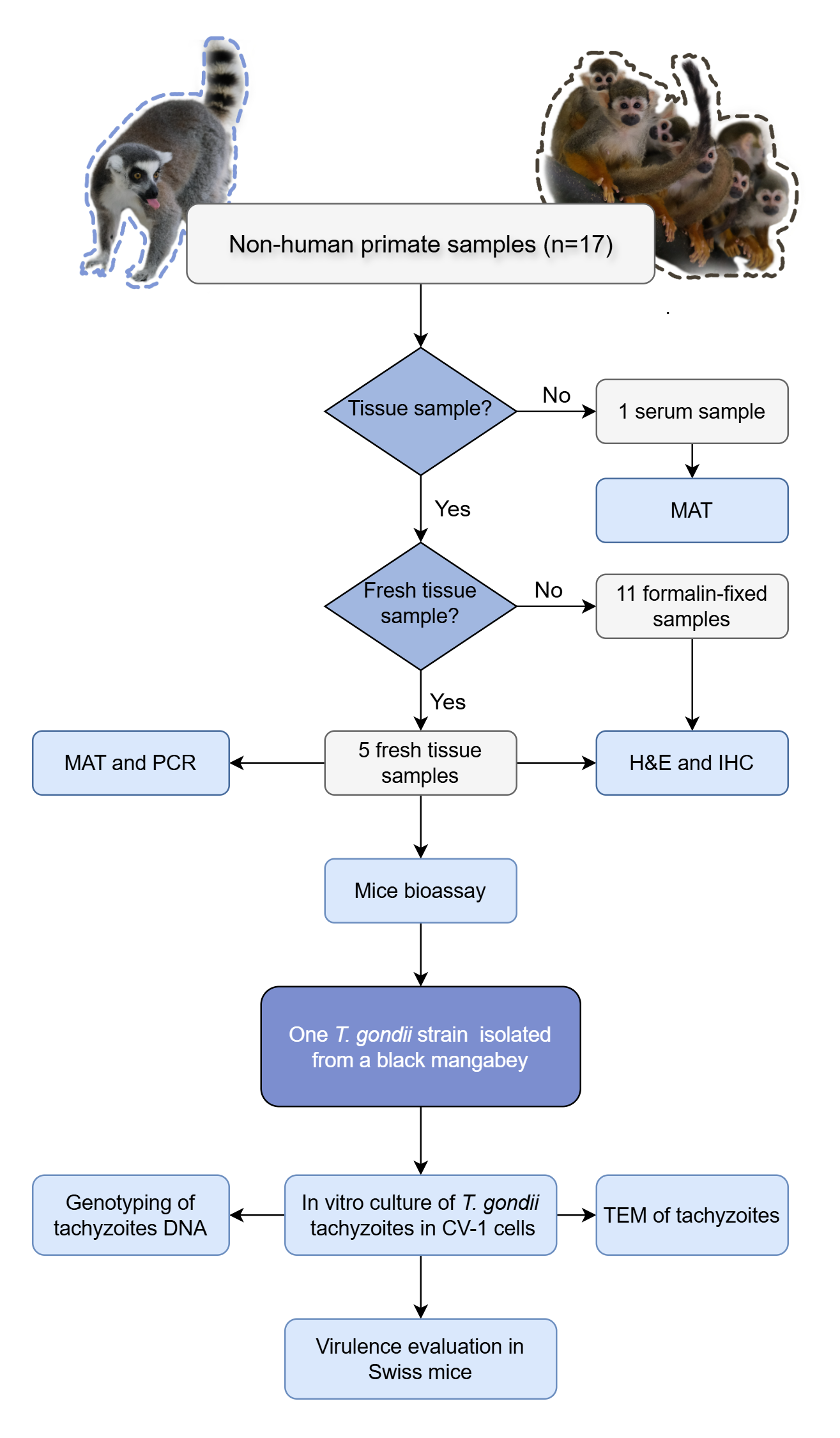

Supplement: S1 Fig — (TIF) [file pntd.0013133.s001.tif]

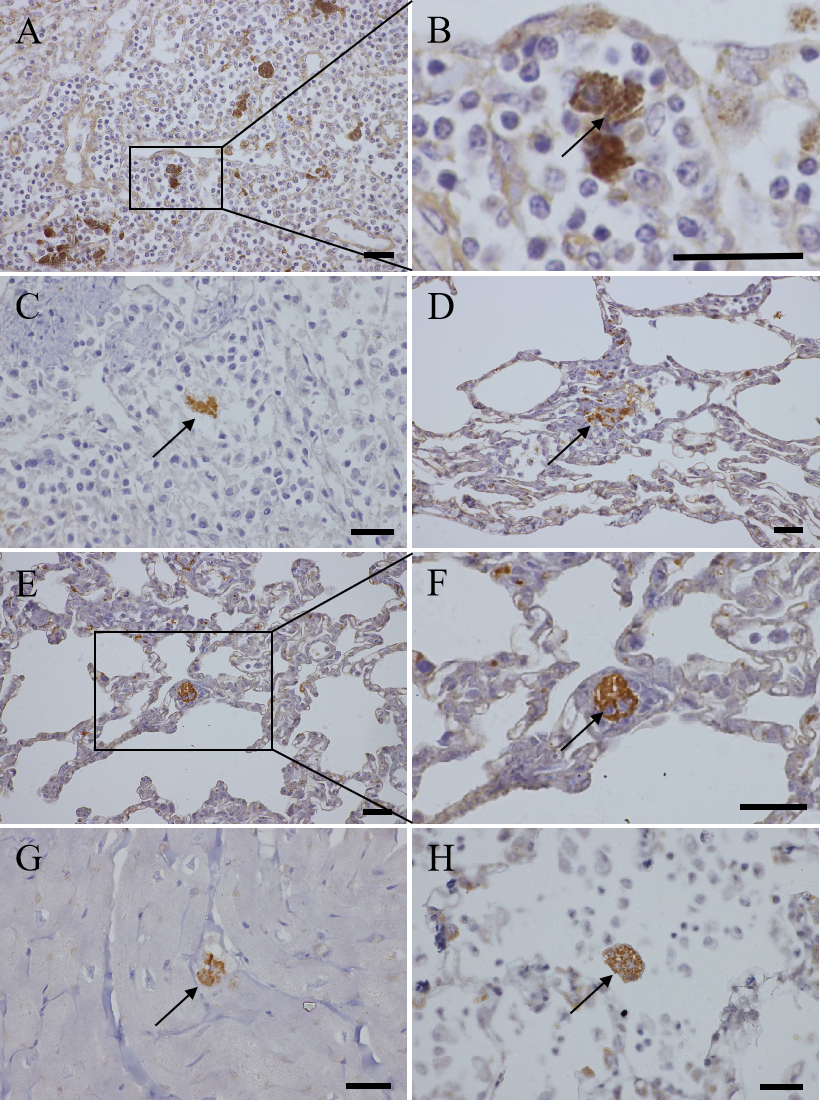

Supplement: S2 Fig — (A, B) Numerous T. gondii tachyzoites (arrow) are enclosed in parasitophorous vacuoles, spleen, case #29. (C) Focally distributed T. gondii tachyzoites (arrow) in the spleen of case #30. (D, E, F) T. gondii tachyzoites gathered in the interstitium of the lung (arrow) in case #33. (G) T. gondii cysts in cardiac fibers with a nearly elliptical shape, case #35. (H) The shed cell in the alveolar cavity was filled with T. gondii tachyzoites, case #41. Primary antibody: polyclonal rabbit anti-T. gondii antibody. Secondary antibody: anti-rabbit IgG conjugated with HRP/DAB. Bar = 50 μm. (TIF) [file pntd.0013133.s002.tif]

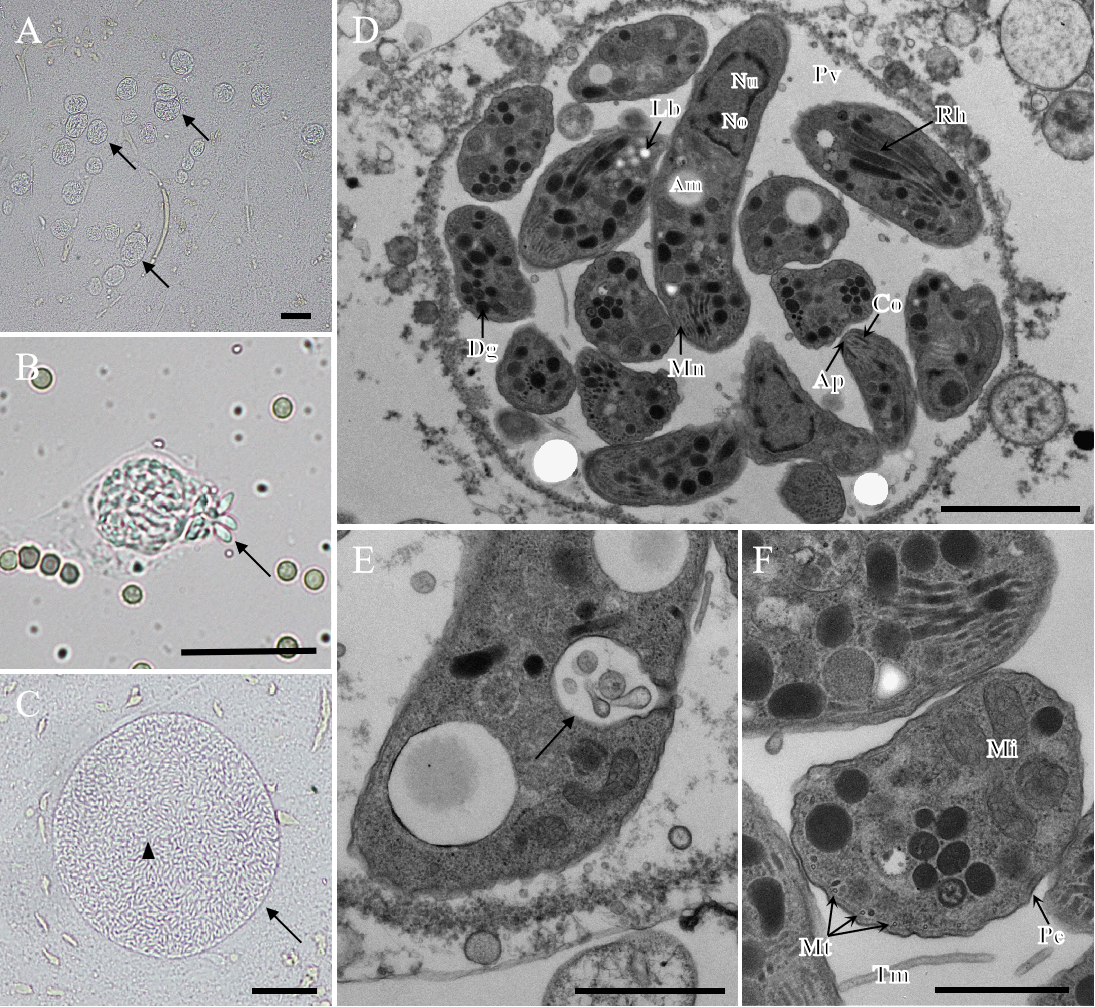

Supplement: S3 Fig — (A) Dozens of T. gondii cysts (arrow) in the brains of Swiss mice (Tox#20–53, M#997) 30 days post-inoculation, smear, unstained, bar = 50 μm. (B) A parasitophorous vacuole ruptured to release several tachyzoites (arrow), lung, Swiss mouse (Tox#20–54, M#7), 15 days post-inoculation, smear, unstained, bar = 50 μm. (C) Hundreds of T. gondii bradyzoites (triangle) enclosed in a thin cyst wall (arrow), Swiss mouse (Tox#20–55, M#21) brain, 112 days post-inoculation, smear, unstained, bar = 50 μm. (D) Several tachyzoites enclosed in a parasitophorous vacuole (Pv), cell cultures. The apical ring (Ap), conoid (Co), nucleus (Nu), nucleolus (No), rhoptries (Rh), micronemes (Mn), lipid body (Lb), dense granules (Dg), and amylopectin (Am) are visible. TEM, bar = 2 μm. (E) A vesicle in exocytosis, presumably containing tubulovesicular membranes (arrow). TEM, bar = 1 μm. (F) In the transverse section of tachyzoites, well-developed mitochondria (Mi), regularly arranged microtubules (Mt), and pellicles (Pe) are visible. The tubulovesicular membranes (Tm) are located around the tachyzoites. TEM, bar = 500 nm. (TIF) [file pntd.0013133.s003.tif]

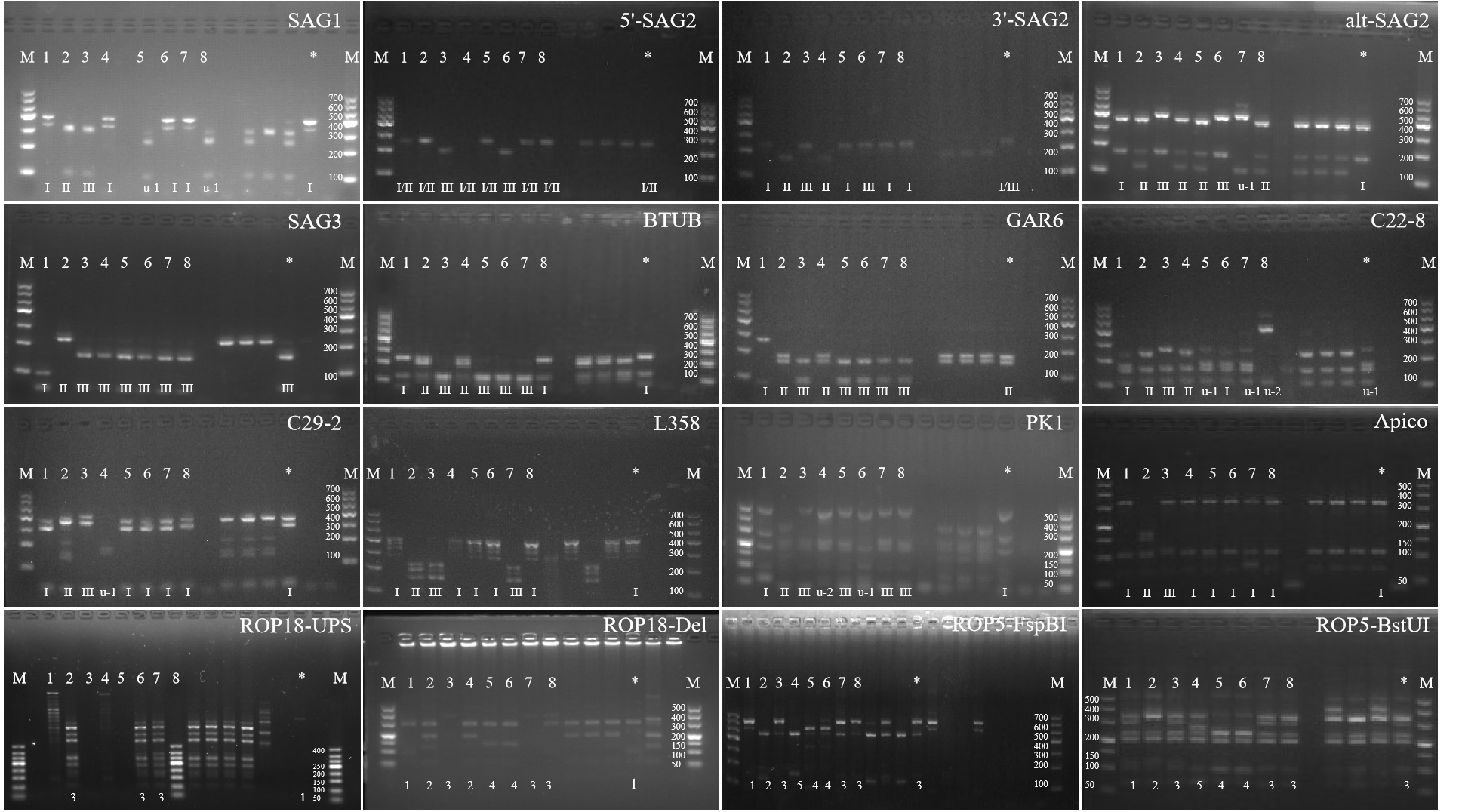

Supplement: S4 Fig — 1: GT1, 2: PTG, 3: CTG, 4: TgCgCal, 5: MAS, 6: TgCatBr5, 7: TgCatBr64, 8: TgToucan (TgRsCr1), *: TgMonkeyCHn3, and M: Marker. (TIF) [file pntd.0013133.s004.tif]
