# Supplementary material for: First isolation of viable Toxoplasma gondii from a black mangabey (Lophocebus aterrimus) reveals the emergence of the Africa 1 lineage in East Asia
Source: PLoS Negl Trop Dis. 2025 Jul 23;19(7):e0013133. doi: 10.1371/journal.pntd.0013133 (PMC12286360; doi:10.1371/journal.pntd.0013133)
Supplement: S5 Fig — M: Makers; 1: GT1; 2: PTG; 3: CTG; 4: MAS; 5: TgCgCa1; 6: TgCatBr5; 7: TgCatBr64; 8: TgRsCr1; *: TgMonkeyCHn3. (PPTX) [file pntd.0013133.s005.pptx]

## Slide 1
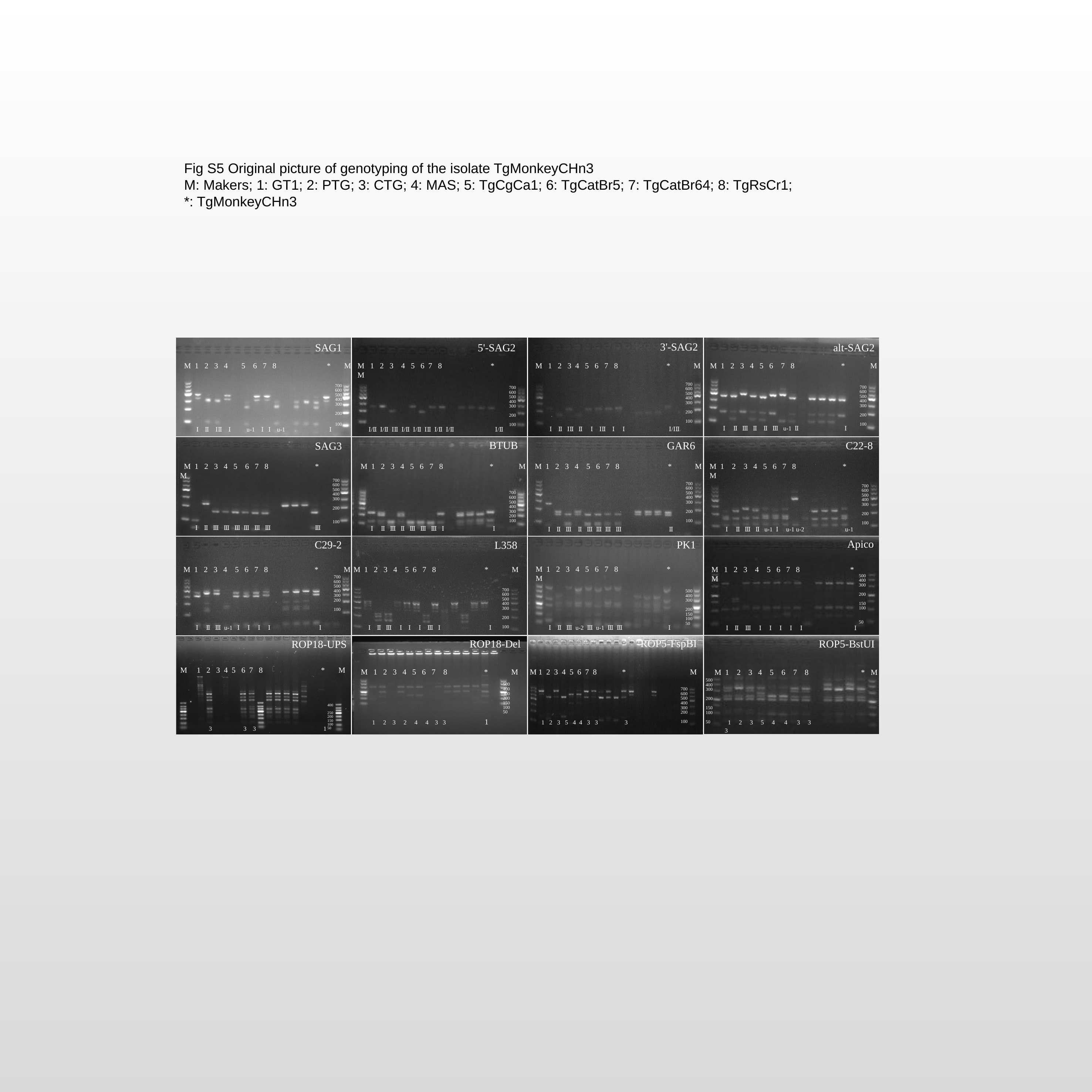

Fig S5 Original picture of genotyping of the isolate TgMonkeyCHn3
M: Makers; 1: GT1; 2: PTG; 3: CTG; 4: MAS; 5: TgCgCa1; 6: TgCatBr5; 7: TgCatBr64; 8: TgRsCr1;
*: TgMonkeyCHn3
3'-SAG2
SAG1
5'-SAG2
alt-SAG2
 M 1 2 3 4 5 6 7 8 * M
M 1 2 3 4 5 6 7 8 * M
M 1 2 3 4 5 6 7 8 * M
M 1 2 3 4 5 6 7 8 * M
700
600
500
400
300
200
100
700
600
500
400
300
200
100
700
600
500
400
300
200
100
700
600
500
400
300
200
100
 Ⅰ Ⅱ ⅠⅡ Ⅱ Ⅰ ⅠⅡ Ⅰ Ⅰ Ⅰ/ⅠⅡ
 Ⅰ Ⅱ Ⅲ Ⅱ Ⅱ Ⅲ u-1 Ⅱ Ⅰ
Ⅰ Ⅱ ⅠⅡ Ⅰ u-1 Ⅰ Ⅰ u-1 Ⅰ
 Ⅰ/Ⅱ Ⅰ/Ⅱ ⅠⅡ Ⅰ/Ⅱ Ⅰ/Ⅱ ⅠⅡ Ⅰ/Ⅱ Ⅰ/Ⅱ Ⅰ/Ⅱ
BTUB
GAR6
C22-8
SAG3
 M 1 2 3 4 5 6 7 8 * M
M 1 2 3 4 5 6 7 8 * M
M 1 2 3 4 5 6 7 8 * M
M 1 2 3 4 5 6 7 8 * M
700
600
500
400
300
200
100
700
600
500
400
300
200
100
700
600
500
400
300
200
100
700
600
500
400
300
200
100
 Ⅰ Ⅱ Ⅲ Ⅱ Ⅲ Ⅲ Ⅲ Ⅰ Ⅰ
 Ⅰ Ⅱ Ⅲ Ⅱ Ⅲ Ⅲ Ⅲ Ⅲ Ⅱ
 Ⅰ Ⅱ Ⅲ Ⅲ Ⅲ Ⅲ Ⅲ Ⅲ Ⅲ
 Ⅰ Ⅱ Ⅲ Ⅱ u-1 Ⅰ u-1 u-2 u-1
Apico
PK1
M 1 2 3 4 5 6 7 8 * M
500
400
300
200
150
100
50
 Ⅰ Ⅱ Ⅲ u-2 Ⅲ u-1 Ⅲ Ⅲ Ⅰ
 Ⅰ Ⅱ Ⅲ Ⅰ Ⅰ Ⅰ Ⅰ Ⅰ Ⅰ
C29-2
L358
 M 1 2 3 4 5 6 7 8 * M
M 1 2 3 4 5 6 7 8 * M
M 1 2 3 4 5 6 7 8 * M
500
400
300
200
150
100
50
700
600
500
400
300
200
100
700
600
500
400
300
200
100
 Ⅰ Ⅱ Ⅲ u-1 Ⅰ Ⅰ Ⅰ Ⅰ Ⅰ
 Ⅰ Ⅱ Ⅲ Ⅰ Ⅰ Ⅰ Ⅲ Ⅰ Ⅰ
ROP5-FspBI
ROP18-Del
ROP5-BstUI
ROP18-UPS
M 1 2 3 4 5 6 7 8 * M
M 1 2 3 4 5 6 7 8 * M
M 1 2 3 4 5 6 7 8 * M
M 1 2 3 4 5 6 7 8 * M
500
400
300
200
150
100
50
500
400
300
200
150
100
50
700
600
500
400
300
200
100
400
250
200
150
100
50
 1 2 3 5 4 4 3 3 3
 1 2 3 5 4 4 3 3 3
 1 2 3 2 4 4 3 3 1
 3 3 3 1
