# Supplementary material for: First isolation of viable Toxoplasma gondii from a black mangabey (Lophocebus aterrimus) reveals the emergence of the Africa 1 lineage in East Asia
Source: PLoS Negl Trop Dis. 2025 Jul 23;19(7):e0013133. doi: 10.1371/journal.pntd.0013133 (PMC12286360; doi:10.1371/journal.pntd.0013133)
Supplement: S1 Table — NA: not available; ND: not done; F: female; M: male; Fixed: formalin fixation; MAT: modified agglutination test; PCR: polymerase chain reaction; IHC: immunohistochemistry; Sp: spleen; L: lung; H: heart. (DOCX) [file pntd.0013133.s006.docx]

**S1 Table Background and *Toxoplasma gondii* infection in non-human primates from zoos (2022–2023, n = 17).**

| Case No. | Received Date | Sample type | Species | Sex, Age | Pathology No. | Clinical Signs and pathological findings | *T. gondii* | | | Mice  Bioassay  (infected/all) |
| --- | --- | --- | --- | --- | --- | --- | --- | --- | --- | --- |
|  |  |  |  |  |  |  | MAT  titer | PCR  positive  tissues | IHC  positive  tissues |  |
| New World non-human primates | | | | | | | | | | |
| Case#29 | 12/6/2022 | Fixed | Red-backed bearded Saki (*Chiropotes chiropotes*) | F, Adult | 3494 | Oronasal hemorrhage. Lung blood vessel rupture. Lung tumor, tumor metastasis to spleen and kidney, and sarcocystis cysts infection in esophagus. | NA | NA | Sp | NA |
| Case#30 | 6/4/2023 | Fresh | Black-capped Capuchin (*Sapajus apella*) | M, Adult | 3558 | Emaciation, dehydration. Suppurative pneumonia, necrotic splenitis, focal liver necrosis, and glomerulonephritis. | 1:1,600 | - | Sp | 0/4 Swiss  0/1 IFN-γ^-/-^ |
| Case#31 | 9/15/2023 | Fixed | Squirrel monkey (*Saimiri sciureus*) | M, Adult | 3609 | Sepsis. Interstitial pneumonia, non-suppurative encephalitis, necrotic splenitis, acute hepatitis, and acute glomerulonephritis. | NA | NA | - | NA |
| Case#32 | 9/15/2023 | Fixed | Squirrel monkey (*Saimiri sciureus*) | F, Adult | 3610 | Emesis after exercise in hot weather. Necrotic myocarditis. gastrorrhagia, non-suppurative encephalitis, acute hepatitis, and interstitial pneumonia. | NA | NA | - | NA |
| Case#33 | 9/19/2023 | Fixed | Squirrel monkey (*Saimiri sciureus*) | NA, Pup | 3619 | Suckling baby. Viremia, interstitial pneumonia, hypoplastic kidneys. | NA | NA | L | NA |
| Case#34 | 9/23/2023 | Fixed | Red-backed bearded Saki (*Chiropotes chiropotes*) | F, Adult | 3621 | Emaciation, mucosal pale. Cecocolic ulcer, spleen amyloidosis, pancreatitis, interstitial pneumonia, and hypoproteinemia. | NA | NA | L, Sp | NA |
| Old World non-human primates | | | | | | | | | | |
| **Case#35** | 1/26/2022 | Fresh | Black mangabey (*Lophocebus aterrimus*) | M, Adult | 3389 | Non-suppurative encephalitis, necrotic splenitis, necrotic pancreatitis, interstitial pneumonia and glomerulonephritis. | 1:32 | H, L | H | 4/4 Swiss |
| Case#36 | 12/6/2022 | Fixed | Patas monkey (*Erythrocebus patas*) | M, Adult | 3493 | Jaundice. Hypoproteinemia, liver capillariasis, whipworm in cecum, necrotic enteritis, and necrotic pancreatitis. | NA | NA | - | NA |
| Case#37 | 3/29/2023 | Fixed | Northern Pig-tailed macaque (*Macaca leonina*) | F, Adult | 3523 | Oronasal hemorrhage. Jaundice.  Cardiac insufficiency, septicemic spleen. | NA | NA | - | NA |
| Case#38 | 3/29/2023 | Fixed | Golden snub-nosed monkey (*Rhinopithecus roxellana*) | F, Pup | 3524 | Cough. Hypoplastic kidneys and liver. Necrotic myocarditis, acute enteritis, interstitial pneumonia. | NA | NA | - | NA |
| Case#39 | 3/29/2023 | Fixed | Rhesus macaque (*Macaca mulatta*) | M, Adult | 3525 | Emaciation, skin wounds, cutaneous dropsy.  Lower urinary tract obstruction, acute renal failure, and lungworm infection. | NA | NA | - | NA |
| Case#40 | 12/25/2023 | Fresh | Black mangabey (*Lophocebus aterrimus*) | F, Adult | 3674 | Female mate of case #35. Jaundice. Viral hepatitis, Catarrhal enteritis, glomerulonephritis, and myocardial necrosis. | <1:2 | **-** | **-** | 0/4 Swiss  0/1 IFN-γ^-/-^ |
| Lemuriformes | | | | | | | | | | |
| Case#41 | 2/8/2023 | Fresh | Ring-tailed lemurs (*Lemur catta*) | NA, Adult | 3509 | Emaciation. Fungal infection, granulomas in multiple organs. | <1:2 | - | L | 0/5 Swiss  0/2 IFN-γ^-/-^ |
| Case#42 | 8/30/2023 | Fixed | Ring-tailed lemurs (*Lemur catta*) | M, Adult | 3597 | Emaciated, depressed, died 20 days post-therapy.  Fibrinous pneumonia, gallstones, extrahepatic cholestasis, intestinal ulcers, fatty liver, necrotic splenitis, and glomerulonephritis. | NA | NA | - | NA |
| Hominoidea | | | | | | | | | | |
| Case#43 | 2/2/2023 | Serum | Orangutan (*Pongo pygmaeus*) | F, 5 years | NA | Healthy. | 1:8,192 | NA | NA | NA |
| Case#44 | 2/12/2023 | Fresh | Chimpanzee (*Pan troglodytes*) | F,31 years | 3510 | Anorexia, dyspnea, weakness, edema, myocardial enzyme, troponin, brain polypeptide, and carcinoembryonic antigen increased. Liver tumor, ovarian tumor, uterine fibroid, cardiac insufficiency, left heart infarction, renal failure, and systemic arterial sclerosis. Hypoproteinemia. | <1:2 | - | - | 0/4 Swiss  0/1 IFN-γ^-/-^ |
| Case#45 | 11/20/2023 | Fixed | Silvery Gibbon (*Hylobates moloch*) | M, 20 days | 3653 | Anorexia, hypothermia, dyspnea.  Suppurative pneumonia, necrotic hepatitis, hypoplastic kidneys. | NA | NA | - | NA |
| Summary (n=17) |  |  |  |  |  |  | 3/6 | 1/5 | 6/16 | 1/5 |

NA: not available; “-”: negative;

F: female; M: male;

Fixed: formalin fixation;

MAT: modified agglutination test;

PCR: polymerase chain reaction;

IHC: immunohistochemistry;

Sp: spleen; L: lung; H: heart.
