# Supplementary material for: First isolation of viable Toxoplasma gondii from a black mangabey (Lophocebus aterrimus) reveals the emergence of the Africa 1 lineage in East Asia
Source: PLoS Negl Trop Dis. 2025 Jul 23;19(7):e0013133. doi: 10.1371/journal.pntd.0013133 (PMC12286360; doi:10.1371/journal.pntd.0013133)
Supplement: S2 Table — (DOCX) [file pntd.0013133.s007.docx]

**S2 Table Genotyping of** ***Toxoplasma gondii*** **TgMonkeyCHn3 strain by PCR-RFLP**

| **Strain ID** | **ToxoDB genotype** | **SAG1** | **5’-3’SAG2** | **Alt.SAG2** | **SAG3** | **BTUB** | **GRA6** | **C22-8** | **C29-2** | **L358** | **PK1** | **Apico** | **ROP18** | **ROP5** |
| --- | --- | --- | --- | --- | --- | --- | --- | --- | --- | --- | --- | --- | --- | --- |
| GT1 (reference) | #10 | I | I | I | I | I | I | I | I | I | I | I | 1 | 1 |
| PTG (reference) | #1 | II/III | II | II | II | II | II | II | II | II | II | II | 2 | 2 |
| CTG (reference) | #2 | II/III | III | III | III | III | III | III | III | III | III | III | 3 | 3 |
| TgCgCa1 (reference) | #66 | I | II | II | III | II | II | II | u-1 | I | u-2 | I | 2 | 5 |
| MAS (reference) | #17 | u-1 | I | II | III | III | III | u-1 | I | I | III | I | 4 | 4 |
| TgCatBr5 (reference) | #19 | I | III | III | III | III | III | I | I | I | u-I | I | 4 | 4 |
| TgCatBr64 (reference) | #111 | I | I | u-1 | III | III | III | u-1 | I | III | III | I | 3 | 3 |
| TgRsCr1 (reference) | #52 | u-1 | I | II | III | I | III | u-2 | I | I | III | I | 3 | 3 |
| TgMonkeyCHn3  (this study) | #6 | I | I | I | III | I | II | u-1 | I | I | I | I | 1 | 3 |
