# Supplementary material for: First isolation of viable Toxoplasma gondii from a black mangabey (Lophocebus aterrimus) reveals the emergence of the Africa 1 lineage in East Asia
Source: PLoS Negl Trop Dis. 2025 Jul 23;19(7):e0013133. doi: 10.1371/journal.pntd.0013133 (PMC12286360; doi:10.1371/journal.pntd.0013133)
Supplement: S3 Table — 10-fold serial dilution concentrations from 1 × 10^4^ to <1 T. gondii tachyzoite/mL were prepared using sterile PBS. Each group of Swiss mice (n = 5) was inoculated by intraperitoneal injection with 1 mL of tachyzoites at different dilutions. T. gondii cysts were found only in one mouse’s brain (n = 80, 23 DPI) from group 102 tachyzoites. DPI: Days post infection. (DOCX) [file pntd.0013133.s008.docx]

**S3 Table Virulence evaluation of** ***Toxoplasma gondii* TgMonkeyCHn3 strain in Swiss mice.**

| Inoculation dose  of tachyzoites | No. infected /  No. inoculated | No. dead /  No. infected | Survival time (DPI) /  No. mice |
| --- | --- | --- | --- |
| 10^4^ | 5/5 | 5/5 | 8 /1, 9 /1, 11 /3 |
| 10^3^ | 5/5 | 5/5 | 9 /2, 10 /2, 13 /1 |
| 10^2^ | 4/5 | 4/4 | 10 /1, 11 /1, 14 /1,  23 /1, ≥60 /1 |
| 10^1^ | 4/5 | 1/4 | ≥60 /4,  10 /1 |
| 1 | 3/5 | 0/3 | ≥ 60 /5 |
| <1 | 0/5 | 0/0 | ≥ 60 /5 |
| Blank control | 0 | 0 | ≥ 60 /5 |

10-fold serial dilution concentrations from 1 × 10^4^ to <1 *T. gondii* tachyzoite/mL were prepared using sterile PBS. Each group of Swiss mice (n = 5) was inoculated by intraperitoneal injection with 1 mL of tachyzoites at different dilutions. *T. gondii* cysts were found only in one mouse's brain (n = 80, 23 DPI) from group 10^2^ tachyzoites. DPI: Days post infection.
