# Supplementary material for: First isolation of viable Toxoplasma gondii from a black mangabey (Lophocebus aterrimus) reveals the emergence of the Africa 1 lineage in East Asia
Source: PLoS Negl Trop Dis. 2025 Jul 23;19(7):e0013133. doi: 10.1371/journal.pntd.0013133 (PMC12286360; doi:10.1371/journal.pntd.0013133)
Supplement: S4 Table — NA: Not available. ND: Not done due to the small sample size. (DOCX) [file pntd.0013133.s009.docx]

**S4 Table Global distribution of *Toxoplasma gondii* ToxoDB genotype #6 strains from animals and comparison of survival time in mice with TgMonkeyCHn3 (1992-2025)**

| **Year** | **Country** | **Species** | **Number** | **Strain name** | ***P* value** ^a^ | **Reference** |
| --- | --- | --- | --- | --- | --- | --- |
| 2025 | China | Black Mangabey | 1 | TgMonkeyCHn3 | \ | This study |
| 2021 | Benin | Chicken,  Mallard duck | 36 | TgA119001-002, TgA11904-037 | Pool, 0.0131*^b^ | [1] |
| 2008 | Brazil | Capybaras | 1 | TgBrCp14 | 0.8293^b^ | [2, 3] |
| 2004 |  | Cat | 5 | TgCatBr2, 12, 17, 21, 30 | TgCatBr17 (0.4482)^b^,  TgCatBr21 (0.4482)^b^,  TgCatBr30 (0.2465)^b^ | [4, 5] |
| 2006 |  |  | 8 | TgCatBr42, 47, 53-55, 62, 71, 75 | TgCatBr42, 53, 55,  and 62 (>0.9999)^b^,  TgCatBr71 (0.6360)^b^  TgCatBr71 (0.0800)^c^ | [6-8] |
| 2012 |  | Chicken | 4 | TgCkBr265, 273, 277, 281 | TgCkBr265 (0.6951)^b^,  TgCkBr273 (0.7964)^b^, TgCkBr281 (0.6302)^b^ | [9, 10] |
| 2006 |  |  | 2 | CH4, CH5 | CH4 (0.0889),  CH5 (0.0719)^c^ | [11, 12] |
| 2007 |  |  | 1 | TgCkBr144 | ND | [13] |
| 2011 |  |  | 3 | TgCkBr201, 203, 207 | TgCkBr201 (0.0132*)^b^, TgCkBr203 (0.2818)^b^, TgCkBr207 (0.0412*)^b^ | [14] |
| 2003 |  |  | 4 | TgCkBr98, 101, 102, 104 | TgCkBr98 (0.8937)^b^,  TgCkBr102 (>0.9)^b^,  TgCkBr104 (0.6294)^b^ | [15, 16] |
| 2018 |  |  | 2 | TgCkBrPr2, 3 | TgCkBrPr2 (0.0320*)^b^, TgCkBrPr3 (0.3701)^b^ | [17] |
| 2003 |  |  | 4 | TgCkBr55, 79, 86, 87 | NA | [16, 18] |
| 2006 |  |  | 2 | TgCkBr123, 124 | TgCkBr123 (0.5624)^b^,  TgCkBr124 (>0.9999)^b^ | [16, 19] |
| 2016 |  |  | 1 | TgCkBrMA4 | ND | [20] |
| 2016 |  |  | 1 | TgChBrUD2 | 0.0062^*c^ | [21] |
| 2002 |  |  | 1 | TgCkBr10 | NA | [16, 22] |
| 2018 |  |  | 2 | TgCkBrEs4, TgCkBrEs5 | NA | [23] |
| 2022 |  |  | 4 | TgCkBrMT40, 42, 49, 51 | NA | [24] |
| 2007 |  | Dog | 2 | TgDgBr3, 7 | NA | [25] |
| 2014 |  |  | 1 | TgDgBr20 | 0.0009*^c^ | [26] |
| 2020 |  |  | 1 | TgDgBrMT2 | NA | [27] |
| 2014 |  | Eared dove | 1 | TgDoveBr7 | NA | [28] |
| 2009 |  | Goat | 4 | TgGtBr2- 4, 9 | TgGtBr3(>0.9999)^b^, TgGtBr4(>0.9999)^b^ | [29, 30] |
| 2008 |  | Sheep | 4 | TgShBr8-11 | TgShBr9 (0.5465)^b^ | [30, 31] |
| 2021 |  | Wagner’s bonneted bat | 1 | NA | NA | [32] |
| 2021 |  | Wild boars | 1 | TgJav1 | ND | [33] |
| 2019 | Senegal | Chicken, Guinea fowl, Muscovy duck, Francolin | 13 | TgA117010, 47, 56-58, 64, 68-72, 74, 77 | NA | [34] |
| 2019 |  | Gambian pouched rat, African giant shrew | 2 | TgCgamb02, TgColiv01 | NA | [35] |
| 2014 | Turkey | Cat | 1 | TgCatTr_Izmir 4 | NA | [36] |
| 2011 | USA | Tammar wallaby | 1 | TgWyUs4 | ND | [37] |
| 2010 | Gabon | Chicken | 11 | GAB5-2007-GAL-DOM1, 2; GAB3-2007-GAL-DOM14, 15, 5B, 19;  GAB3-2007-GAL-DOM1, 2-4, 11 | ND | [38] |
| Total | 7 countries | 16 species | 125 strains | Brazil (n=60), Benin (n=36), Senegal (n=15), Gabon (n=11),  Turkey (n=1), USA (n=1), China (n=1) | | |

^a^: Comparing survival times of infected mice between TgMonkeyCHn3 and *T. gondii* ToxoDB genotype #6 strains using the asymptotic log-rank test for pair groups.

^b^: The mouse survival times in bioassay of the strain compared with overall survival times (100-104 tachyzoites) of TgMonkeyCHn3.

^c^: The mouse survival times of the strain compared with TgMonkeyCHn3 at the same or very close dose of tachyzoites.

NA: Not available.

ND: Not done due to the small sample size.

**References：**

1. Hamidović A, Etougbétché JR, Tonouhewa ABN, Galal L, Dobigny G, Houémènou G, et al. A hotspot of *Toxoplasma gondii* Africa 1 lineage in Benin: how new genotypes from West Africa contribute to understand the parasite genetic diversity worldwide. PLoS Negl Trop Dis. 2021; 15(2):e0008980.

2. Yai LE, Ragozo AM, Aguiar DM, Damaceno JT, Oliveira LN, Dubey JP, et al. Isolation of *Toxoplasma gondii* from capybaras (*Hydrochaeris hydrochaeris*) from São Paulo State, Brazil. J Parasitol. 2008; 94(5):1060-3.

3. Yai LE, Ragozo AM, Soares RM, Pena HF, Su C, Gennari SM. Genetic diversity among capybara (*Hydrochaeris hydrochaeris*) isolates of *Toxoplasma gondii* from Brazil. Vet Parasitol. 2009; 162(3-4):332-7.

4. Dubey JP, Navarro IT, Sreekumar C, Dahl E, Freire RL, Kawabata HH, et al. *Toxoplasma gondii* infections in cats from Paraná, Brazil: seroprevalence, tissue distribution, and biologic and genetic characterization of isolates. J Parasitol. 2004; 90(4):721-6.

5. Su C, Zhang X, Dubey JP. Genotyping of *Toxoplasma gondii* by multilocus PCR-RFLP markers: a high resolution and simple method for identification of parasites. Int J Parasitol. 2006; 36(7):841-8.

6. Pena HF, Gennari SM, Dubey JP, Su C. Population structure and mouse-virulence of *Toxoplasma gondii* in Brazil. Int J Parasitol. 2008; 38(5):561-9.

7. Pena HF, Soares RM, Amaku M, Dubey JP, Gennari SM. *Toxoplasma gondii* infection in cats from São Paulo state, Brazil: seroprevalence, oocyst shedding, isolation in mice, and biologic and molecular characterization. Res Vet Sci. 2006; 81(1):58-67.

8. Hamilton CM, Black L, Oliveira S, Burrells A, Bartley PM, Melo RPB, et al. Comparative virulence of Caribbean, Brazilian and European isolates of *Toxoplasma gondii*. Parasit Vectors. 2019; 12(1):104.

9. Beltrame MA, Pena HF, Ton NC, Lino AJ, Gennari SM, Dubey JP, et al. Seroprevalence and isolation of *Toxoplasma gondii* from free-range chickens from Espírito Santo state, southeastern Brazil. Vet Parasitol. 2012; 188(3-4):225-30.

10. Pena HF, Vitaliano SN, Beltrame MA, Pereira FE, Gennari SM, Soares RM. PCR-RFLP genotyping of *Toxoplasma gondii* from chickens from Espírito Santo state, Southeast region, Brazil: new genotypes and a new SAG3 marker allele. Vet Parasitol. 2013; 192(1-3):111-7.

11. Brandão GP, Ferreira AM, Melo MN, Vitor RW. Characterization of *Toxoplasma gondii* from domestic animals from Minas Gerais, Brazil. Parasite. 2006; 13(2):143-9.

12. Silva LA, Andrade RO, Carneiro AC, Vitor RW. Overlapping *Toxoplasma gondii* genotypes circulating in domestic animals and humans in Southeastern Brazil. PLoS One. 2014; 9(2):e90237.

13. Dubey JP, Sundar N, Gennari SM, Minervino AH, Farias NA, Ruas JL, et al. Biologic and genetic comparison of *Toxoplasma gondii* isolates in free-range chickens from the northern Pará state and the southern state Rio Grande do Sul, Brazil revealed highly diverse and distinct parasite populations. Vet Parasitol. 2007; 143(2):182-8.

14. Soares RM, Silveira LH, da Silva AV, Ragozo A, Galli S, Lopes EG, et al. Genotyping of *Toxoplasma gondii* isolates from free range chickens in the Pantanal area of Brazil. Vet Parasitol. 2011; 178(1-2):29-34.

15. Dubey JP, Navarro IT, Graham DH, Dahl E, Freire RL, Prudencio LB, et al. Characterization of *Toxoplasma gondii* isolates from free range chickens from Paraná, Brazil. Vet Parasitol. 2003; 117(3):229-34.

16. Dubey JP, Velmurugan GV, Chockalingam A, Pena HF, de Oliveira LN, Leifer CA, et al. Genetic diversity of *Toxoplasma gondii* isolates from chickens from Brazil. Vet Parasitol. 2008; 157(3-4):299-305.

17. Vieira FEG, Sasse JP, Minutti AF, Miura AC, de Barros LD, Cardim ST, et al. *Toxoplasma gondii*: prevalence and characterization of new genotypes in free-range chickens from south Brazil. Parasitol Res. 2018; 117(3):681-8.

18. Dubey JP, Graham DH, da Silva DS, Lehmann T, Bahia-Oliveira LM. *Toxoplasma gondii* isolates of free-ranging chickens from Rio de Janeiro, Brazil: mouse mortality, genotype, and oocyst shedding by cats. J Parasitol. 2003; 89(4):851-3.

19. Dubey JP, Gennari SM, Labruna MB, Camargo LM, Vianna MC, Marcet PL, et al. Characterization of *Toxoplasma gondii* isolates in free-range chickens from Amazon, Brazil. J Parasitol. 2006; 92(1):36-40.

20. Sousa IC, Pena HF, Santos LS, Gennari SM, Costa FN. First isolation and genotyping of *Toxoplasma gondii* from free-range chickens on São Luis island, Maranhão state, Brazil, with a new genotype described. Vet Parasitol. 2016; 223:159-64.

21. Lopes CS, Franco PS, Silva NM, Silva DA, Ferro EA, Pena HF, et al. Phenotypic and genotypic characterization of two *Toxoplasma gondii* isolates in free-range chickens from Uberlândia, Brazil. Epidemiol Infect. 2016; 144(9):1865-75.

22. Dubey JP, Graham DH, Blackston CR, Lehmann T, Gennari SM, Ragozo AM, et al. Biological and genetic characterisation of *Toxoplasma gondii* isolates from chickens (*Gallus domesticus*) from São Paulo, Brazil: unexpected findings. Int J Parasitol. 2002; 32(1):99-105.

23. Ferreira TCR, Buery JC, Moreira NIB, Santos CB, Costa JGL, Pinto LV, et al. *Toxoplasma gondii*: isolation, biological and molecular characterisation of samples from free-range *Gallus gallus domesticus* from countryside Southeast Brazil. Rev Bras Parasitol Vet. 2018; 27(3):384-9.

24. Witter R, Pena HFJ, Maia MO, da Costa Freitas L, Almeida SLH, de Aguiar DM, et al. First report on the isolation and genotyping of *Toxoplasma gondii* strains from free-range chickens in the state of Mato Grosso, Midwestern Brazil. Comp Immunol Microbiol Infect Dis. 2022; 80:101725.

25. Dubey JP, Gennari SM, Sundar N, Vianna MC, Bandini LM, Yai LE, et al. Diverse and atypical genotypes identified in *Toxoplasma gondii* from dogs in São Paulo, Brazil. J Parasitol. 2007; 93(1):60-4.

26. Pena HF, Moroz LR, Sozigan RK, Ajzenberg D, Carvalho FR, Mota CM, et al. Isolation and biological and molecular characterization of *Toxoplasma gondii* from canine cutaneous toxoplasmosis in Brazil. J Clin Microbiol. 2014; 52(12):4419-20.

27. Witter R, Pena HFJ, Maia MO, de Magalhães AO, Morgado TO, Colodel EM, et al. Isolation and genotyping of *Toxoplasma gondii* in the Midwestern Brazil revealed high genetic diversity and new genotypes. Acta Trop. 2020; 212:105681.

28. Barros LD, Taroda A, Zulpo DL, Cunha IA, Sammi AS, Cardim ST, et al. Genetic characterization of *Toxoplasma gondii* isolates from eared doves (*Zenaida auriculata*) in Brazil. Rev Bras Parasitol Vet. 2014; 23(4):443-8.

29. Ragozo AM, Yai LE, Oliveira LN, Dias RA, Gonçalves HC, Azevedo SS, et al. Isolation of *Toxoplasma gondii* from goats from Brazil. J Parasitol. 2009; 95(2):323-6.

30. Ragozo AM, Pena HF, Yai LE, Su C, Gennari SM. Genetic diversity among *Toxoplasma gondii* isolates of small ruminants from Brazil: novel genotypes revealed. Vet Parasitol. 2010; 170(3-4):307-12.

31. Ragozo AM, Yai RL, Oliveira LN, Dias RA, Dubey JP, Gennari SM. Seroprevalence and isolation of *Toxoplasma gondii* from sheep from São Paulo state, Brazil. J Parasitol. 2008; 94(6):1259-63.

32. Cabral AD, Su C, Soares RM, Gennari SM, Sperança MA, da Rosa AR, et al. Occurrence and diversity of Sarcocystidae protozoa in muscle and brain tissues of bats from São Paulo state, Brazil. Int J Parasitol Parasites Wildl. 2021; 14:91-6.

33. Machado DMR, de Barros LD, de Souza Lima Nino B, de Souza Pollo A, Dos Santos Silva AC, Perles L, et al. *Toxoplasma gondii* infection in wild boars (*Sus scrofa*) from the State of São Paulo, Brazil: serology, molecular characterization, and hunter's perception on toxoplasmosis. Vet Parasitol Reg Stud Reports. 2021; 23:100534.

34. Galal L, Sarr A, Cuny T, Brouat C, Coulibaly F, Sembène M, et al. The introduction of new hosts with human trade shapes the extant distribution of *Toxoplasma gondii* lineages. PLoS Negl Trop Dis. 2019; 13(7):e0007435.

35. Galal L, Schares G, Stragier C, Vignoles P, Brouat C, Cuny T, et al. Diversity of *Toxoplasma gondii* strains shaped by commensal communities of small mammals. Int J Parasitol. 2019; 49(3-4):267-75.

36. Can H, Döşkaya M, Ajzenberg D, Özdemir HG, Caner A, İz SG, et al. Genetic characterization of *Toxoplasma gondii* isolates and toxoplasmosis seroprevalence in stray cats of İzmir, Turkey. PLoS One. 2014; 9(8):e104930.

37. Dubey JP, Velmurugan GV, Rajendran C, Yabsley MJ, Thomas NJ, Beckmen KB, et al. Genetic characterisation of *Toxoplasma gondii* in wildlife from North America revealed widespread and high prevalence of the fourth clonal type. Int J Parasitol. 2011; 41(11):1139-47.

38. Mercier A, Devillard S, Ngoubangoye B, Bonnabau H, Bañuls A-L, Durand P, et al. Additional haplogroups of *Toxoplasma gondii* out of Africa: population structure and mouse-virulence of strains from Gabon. PLoS Negl Trop Dis. 2010; 4(11): e876.
