# Supplementary material for: First isolation of viable Toxoplasma gondii from a black mangabey (Lophocebus aterrimus) reveals the emergence of the Africa 1 lineage in East Asia
Source: PLoS Negl Trop Dis. 2025 Jul 23;19(7):e0013133. doi: 10.1371/journal.pntd.0013133 (PMC12286360; doi:10.1371/journal.pntd.0013133)
Supplement: Attachment — Submitted filename: 3 Response to Reviewers 0325.docx [file pntd.0013133.s012.docx]

PNTD-D-25-00020

Dear Editors and Reviewers,

We sincerely appreciate the reviewers' insightful comments and constructive suggestions. It encourages us to do more research about the topic of *Toxoplasma gondii* and wild animals health. We have attempted to clarify all the questions in the text and colored the revision part (Blue color). We hope to have clarified all the questions. Thank you for your kind help.

**Methods**:

**Reviewer #1**: ok

**Response:** Thank you!

**Reviewer #2:**

Q1: PCR conditions (cycling parameters, reaction mix, controls, primer sequences) are missing. The modified agglutination test (MAT) lacks specificity details, such as: were positive and negative controls included? How was MAT sensitivity validated for NHP samples?

**Response:** we have added to the text about PCR conditions (Page 6, Line 7-15) and MAT details and discussed about MAT validity for NHP (Page 6, Line 1-3). Thank you for your suggestions!

Q2: The methodology states that Swiss mice and IFN-γ knockout mice were used, but does not explain why was IFN-γ knockout used? What were the humane endpoints for euthanasia? The statistical rationale for the number of mice used (n=5 per group) is not justified.

**Response:** We have added an explanation of the reasons for using IFN-γ knockout mice to the text (Page 7, Line 16-18). The humane endpoint of euthanasia was the severe signs exhibited by mice, e.g., respiratory distress, unwillingness to move and eat, neurological symptoms or hypothermia, which have been added in the text (Page 8, Line 1-2).

Thanks for your suggestions! Yes, comparison between mice groups, the statistical rationale for the number of mice used (n=5 per group) is not justified. It needs 6 mice or more mice. The aim of this study is to evaluate the biological characteristics of the new isolated *T. gondii* strain. Considering the aim and 3R principle (reduce, reuse and recycle), a small number of mice were used in this study. In our next study, we will carefully select the number of mice according to statistical rationale, many thanks!

Q3: The IHC methodology lacks specificity details, such as what antibody dilution and staining conditions were used?

**Response:** Thank you for your comments! We have described more clearly the protocol of IHC and some specific details (Page 7, Line 1-9).

Q4: TEM images are provided, but sample preparation details (fixation method, contrast staining) are missing. Also, PCR conditions should included in the text.

**Response:** We have placed TEM method in a new subsection (2.7) and added the details you have raised (Page 9, Line 2-8).

**Reviewer #3:**

Q1: Page 4, Lines 8-9: please give reference for giving seropositivity to 1:8 dilution

**Response:** We appreciate the reviewer's suggestion and we've added references (Page 6, Line 2).

Xin S, Jiang N, Yang L, Zhu N, Huang W, Li J, et al. Isolation, genotyping and virulence determination of a Toxoplasma gondii strain from non-human primate from China. Transbound Emerg Dis. 2022; 69(2):919-25.

Yang L, Ren H, Zhu N, Xin S, Mao G, Ma Y, et al. Isolation and Genetic Characterization of Toxoplasma gondii from a Patas Monkey (Erythrocebus patas) in China. Genes (Basel). 2023; 14(8).

Q2: page 4, Lines 11-14: Which tisues were used, how were the tissue collected (In terms of how the cross contamination was prevented)?

**Response:** DNA was extracted from all collected tissues including liver, tongue, spleen, kidney, heart, lungs, brain, mesenteric lymph nodes, pancreas and skeletal muscle (Page 6, Line 5-7). Separated sterile bags were used for each fresh tissue during autopsy sampling to avoid cross-contamination (Page 5, Line 14-15).

Q3: How was the digestion performed? please describe it. Please give details about DNA extraction? PCR method has to be described in detail.

**Response:** Thank you for your comments! We have added more detailed protocols about digestion (Page 7, Line 12-20), DNA extraction and PCR (Page 6, Line 6-15) in the methods section.

Q4: Page 4, Line 19: what is the source of antibody?

**Response:**

The primary antibody was rabbit anti-*T. gondii* polyclonal antibody provided by Dr. J. P. Dubey (Beltsville, MD, USA Agricultural Research Service, USDA). The secondary antibody was included in the rabbit-specific HRP/DAB IHC detection kit was purchased from Abcam (ab64264). We have clarified the above information in the revised text (Page 6, Line 20-22; Page 7, Line 1-2).

Further, corresponding Author (Yurong Yang) had worked in Dr. Dubey Lab during 2013.9-2014.9. Dr. Dubey and Yurong Yang also have co-published papers (n=9, 2014-2025).

[1] **Yang Y**, Zhang Q, Kong Y, Ying Y, Kwok OC, Liang H, **Dubey JP**. Low prevalence of Neospora caninum and Toxoplasma gondii antibodies in dogs in Jilin, Henan and Anhui Provinces of the People's Republic of China. BMC Vet Res. 2014 Dec 12; 10:295

[2] **Yang YR**, Ying YQ, Verma SK, Cassinelli AB, Kwok OC, Liang H, Pradhan AK, Zhu XQ, Su CL, **Dubey JP**. Isolation and genetic characterization of viable Toxoplasma gondii from tissues and feces of cats from the central region of China. Vet Parasitol. 2015, 211(3-4): 283-288.

[3] **Yang Y**, Xin S, Murata FHA, Cerqueira-Cézar CK, Kwok OCH, Su C, **Dubey JP**. Recent epidemiologic, clinical, subclinical and genetic diversity of Toxoplasma gondii infections in bats. Res Vet Sci. 2021 Nov;140:193-197.

[4] **Dubey JP**, Murata FHA, Cerqueira-Cézar CK, Kwok OCH, **Yang Y**, Su C. Recent epidemiologic, clinical, and genetic diversity of Toxoplasma gondii infections in non-human primates. Res Vet Sci. 2021 May;136:631-641.

[5] **Dubey JP**, Murata FHA, Cerqueira-Cézar CK, Kwok OCH, **Yang YR**. Public Health Significance of Toxoplasma gondii Infections in Cattle: 2009-2020. J Parasitol. 2020 Nov 12;106(6):772-788.

[6] **Dubey JP**, Cerqueira-Cézar CK, Murata FHA, Kwok OCH, Hill D, **Yang Y**, Su C. All about Toxoplasma gondii infections in pigs: 2009-2020. Vet Parasitol. 2020 Dec;288:109185.

[7] **Dubey JP**, Murata FHA, Cerqueira-Cézar CK, Kwok OCH, **Yang Y**, Su C. Toxoplasma gondii infections in dogs: 2009-2020. Vet Parasitol. 2020 Aug 31;287:109223.

[8] **Dubey JP**, Pena HFJ, Cerqueira-Cézar CK, Murata FHA, Kwok OCH, **Yang YR**, Gennari SM, Su C. Epidemiologic significance of Toxoplasma gondii infections in chickens (Gallus domesticus): the past decade. Parasitology. 2020 Oct;147(12):1263-1289.

[9] **Dubey JP**, Cerqueira-Cézar CK, Murata FHA, Kwok OCH, **Yang YR**, Su C. All about toxoplasmosis in cats: the last decade. Vet Parasitol. 2020 Jul;283:109145.

Q5: Page 5, line 1: did you give tissues directly or digested it before use? please give the protocol.
how did you inoculate the tissues?

**Response:** Tissues were digested before inoculation to mice and Inoculation was performed by subcutaneous injection at a dose of 1 mL per mouse (Page 7, Line 11-20). We have added a more detailed protocol about bioassay.

Q6: Page 5, line 4: Examined by which method?

**Response:** Tissues (lungs, mesenteric lymph nodes, or brains) of dead mice were examined for *T. gondii* parasites by smears microscopically. Please see Page 8, Line 2-4.

Q7: page 5, line 5: did you previously check the mouse for *T. gondii* Ab? Please explain the details of the test to detect *T. gondii* Ab.

**Response:** *T. gondii*-free mice were used in this study, and they were tested to be seronegative prior to the experiment. More details about MAT method were added (Page 5 Line 21- Page 6 Line 3).

Q8: page 5, line 7: please describe the CV-1 cell culture and inoculation method In detail

**Response:** Thank you for your nice suggestions. More details were added, see Page 8, Line8-15.

Q9: page 5, line 9: please give detailed information about multiplex PCR

page 5, line 10-11: Please give details of the method about typing and please give detailed information about the reference strains

**Response:** Thanks for your suggestions! The details were added in text, including genetic markers and reference *T. gondii* strains DNA (Page 8, Line 16-22).

Su C, Shwab EK, Zhou P, Zhu XQ, Dubey JP. Moving towards an integrated approach to molecular detection and identification of Toxoplasma gondii. Parasitology. 2010; 137(1):1-11.

The PCR-RFLP for T. gondii was initially developed by Dr. Su, and corresponding Author (Yurong Yang) had masted this protocol in Dr. Dubey Lab, the details of the method was showed in the following Table.

The genotyping was performed using multilocus PCR-RFLP. Briefly, 10 genetic markers (SAG1, SAG2, SAG3, BTUB, GRA6, c22-8, c29-2, L358, PK1, and Apico) were amplified through multiplex PCR with external primers, followed by nested PCR using marker-specific internal primers. The nested PCR products were treated with restriction enzymes, and the resulting RFLP patterns were analyzed via agarose gel electrophoresis. These patterns were compared to 8 reference strains (GT1, PTG, CTG, MAS, TgCgCa1, TgCatBr5, TgCatBr64 and TgRsCr1) to determine allele types at each locus. The combination of alleles across all 10 markers identified TgMonkeyCHn3 as ToxoDB genotype #6 by comparing with other isolates in the ToxoDB database (https://toxodb.org). This approach ensures high-resolution genotyping by integrating multiplex PCR efficiency and nested PCR sensitivity, validated through established reference controls.

The ROP18 and ROP5 allele type: Briefly, nested PCR amplified target regions of ROP18 (DEL or UPS domains) and ROP5, followed by restriction enzymes treatment. Electrophoresis patterns were compared to 8 reference strains to assign allele types.

In addition, we have supplemented a table and a gel figure of the genotyping results (Table S2, Fig S2).

Q10: page 5, line 12-14: please give detailed information about this method

**Response:** To describe the protocol more clearly, we have placed TEM method in a new subsection (2.7) and added the details. Please see Page 9, Line 2-8.

Q11: please add Kaplan-Meier survival curve to your statistical section

**Response:** Thanks! Kaplan-Meier analysis was added in the statistical section (Page 10, Line 2-4).

**Reviewer #4:**

In this study, serological, molecular, and bioassay methods were used to investigate *T. gondii* infection in 17 captived NHP in zoos from China between 2022 and 2023. In the study, it was stated that the live strain of Toxoplasma gondii was determined for the first time in Black mangabey and that this animal was defined as a new host for *T. gondii*. Molecular characterization of T.gondi was performed.

Q1: However, it was observed that a wide variety of methods were used. Generally, the reference is given without giving the details of these methods.

**Response:** Thanks! Detailed protocols are now provided in the Methods section, including PCR, IHC, MAT, bioassay, in vitro cultivation, genotyping, TEM, virulence evaluation in mice and statistical analysis. We hope that it is now clearer.

Q2: Page 10, Result Line 6 should review the expression "31 December 31".

**Response:** It was revised into “December 31, 2024”. Thank you for your reminder!

Q3: It would be useful to show the workflow in the Method section with a picture.

**Response:** Thanks for your nice suggestions! The workflow (Fig S1) was added.

**Reviewer #5:** (No Response)

**Results**:

**Reviewer #1**: ok

**Response:** Thank you!

**Reviewer #2:**

Q1: The study reports infection rates of 83.3% in New World monkeys vs. 16.7% in Old World monkeys, but: No statistical test (e.g., chi-square test) is used to determine if the difference is significant. Confidence intervals (CIs) are not provided for prevalence rates.Also, the survival curve analysis (Figure 3) lacks statistical validation (e.g., Kaplan-Meier analysis, log-rank test).

**Response:** Thanks for your suggestions! They have been revised.

(1) 2.9 Statistical analysis section now includes log-rank (Mantel-Cox) test and Chi-square test (Page 9, Line 20-22). (2) Statistical significance test was added to Figure 3, Results (Page 12, Line 15-18) and Discussion (Page 17, Line 2-7). (3) 95% confidence intervals were added in the Results.

Q2: The study states that TgMonkeyCHn3 was highly virulent, but does not compare survival outcomes with previously reported ToxoDB #6 strains.

**Response:** Thanks! The mouse survival times of other ToxoDB #6 isolates were added and compared with TgMonkeyCHn3 in Table S4 and Discussion section (Page 17, Line 2-7).

**Reviewer #3:**

Q1: Page 6, lines 6-13: please make a simple group name for mouse other than giving complex names such as Tox# 20-55 etc.

**Response:** After careful consideration, we have retained the original naming convention in revised text for the following reasons: (1) This systematic labeling directly links each group to its corresponding species source (“20” represents the species ID of the NHPs) and experimental batch ("50"), preventing confusion between different groups. (2) This format follows the principle of prior published study (Table 3 in Reference of: Jiang N, et al. Isolation and characterization of *Toxoplasma gondii* from captive caracals (*Caracal caracal*). Int J Parasitol Parasites Wildl. 2020, 13:196-201.).

For ease of understanding, the naming logic was explained in the Methods section 2.5 (Page 7, Line 21- Page 8, Line 1): “Each group was sequentially named Tox#20-x. For example, in “Tox#20-50”, “Tox” represents *T. gondii* bioassay; “20” represents the species ID of the NHPs, and “50” represents the group ID.”

Q2: Please give the details of genotyping, for example show did you reach ToxoDB#6 and gene allele type of ROP18/ROP5 was 3/3.

**Response:** Thanks for your suggestions.

As recommended, each reference *T. gondii* strains name and its DNA source and 10 genetic markers names have been added (Page 8, Line 16-22). In addition, we have supplemented table and figure of the genotyping results (Table S2, Figure S2). However, given that the methods of genotyping have been described in detail in previous studies, we chose not to repeat them to avoid making the article too redundant. Here are the protocols for genotyping:

The genotyping was performed using multilocus PCR-RFLP. Briefly, 10 genetic markers (SAG1, SAG2, SAG3, BTUB, GRA6, c22-8, c29-2, L358, PK1, and Apico) were amplified through multiplex PCR with external primers, followed by nested PCR using marker-specific internal primers. The nested PCR products were treated with restriction enzymes, and the resulting RFLP patterns were analyzed via agarose gel electrophoresis. These patterns were compared to 8 reference strains (GT1, PTG, CTG, MAS, TgCgCa1, TgCatBr5, TgCatBr64 and TgRsCr1) to determine allele types at each locus. The combination of alleles across all 10 markers identified TgMonkeyCHn3 as ToxoDB genotype #6 by comparing with other isolates in the ToxoDB database (https://toxodb.org/toxo/app). This approach ensures high-resolution genotyping by integrating multiplex PCR efficiency and nested PCR sensitivity, validated through established reference controls.

The ROP18 and ROP5 allele type: Briefly, nested PCR amplified target regions of ROP18 (DEL or UPS domains) and ROP5, followed by restriction enzymes treatment. Electrophoresis patterns were compared to 8 reference strains to assign allele types.

Q3: please change figure 3 explanation as “Kaplan-Meier survival curve of Swiss mice infected with various doses of *T. gondii* TgMonkeyCHn3 strain tachyzoites

**Response:** Thanks for your suggestions! It was revised.

Q4: Please cite the data showed on figure 4 in a separate table.

**Response:** Thanks! Data were summarized in Supplemental Table S4.

**Reviewer #4:**

Q1: The analysis results are adequately expressed in tables and figures.

**Response:** Thanks for your encouraging comments!

**Reviewer #5:** (No Response)

**Conclusions**:
**Reviewer #1:** ok

**Response:** Thank you!

**Reviewer #2:**

Q1: Discussion. One major limitation is the missing comparision between other ToxoDB #6 strains and

the one found in this study. How does its virulence compare to other ToxoDB #6 strains isolated in

Africa/Brazil? Does its ROP18/ROP5 (3/3) allele type match other virulent strains? Those questions

needs to be adressed.

**Response:**

Following your suggestion, the comparison of mouse survival times between other ToxoDB #6 strains and TgMonkeyCHn3 were summarized in Table S4.

Among the reported ToxoDB #6 isolates, only TgCatBr71 had complete virulence information, possessing the same ROP18/ROP5 allele type (1/3), and we have compared the survival time of mice by log-rank test in the revised text. Unfortunately, due to the lack of specific data in previous reports (e.g., inoculation dose, average survival time, inoculation route), there are only 69 ToxoDB #6 strains to statistically compare the survival time with TgMonkeyCHn3.The survival times of 39.1% (27/69, CI: 28.47%-50.94%) ToxoDB #6 isolates showed no significant differences with TgMonkeyCHn3, other 60.9% (42/69, CI: 49.06%-71.53%) showed shorter survival time than TgMonkeyCHn3 (*P* < 0.05). Please see Page 17, Line 2-7.

Q2: What is the possible transmission route in the Zoo? Needs more discussion. Any contamination on food? Or water? Or etc.???

**Response:** It was speculated that the source of TgMonkeyCHn3 was most likely attributed to the introduction of zoo animals from Africa, meaning that the black mangabey (case #35) was already infected with this strain in its original habitat.

We have expanded the Discussion (Page 15, Line 14-22，Page 16, Line 1-13), including aerosol transmission, sexual transmission via semen, endogenous transplacental transmission, the role of the feral cats in the zoo, and the heavy rainstorm flooding leading to oocyst contamination. However, the presence of contamination in the feed and water remained unknown and more investigation was needed. Thank you for your valuable suggestions, which will be the focus of our future research!

Q3: What kind of preventions should take in to consideration

**Response:** Thanks for your comment! The revised manuscript now includes more suggestions for preventing *T. gondii* transmission as follows: (1) Conduct a complete investigation of *T. gondii* infection in animals and practitioners in the local zoo. (2) Control the activity of feral cats. (3) Provide effective *T. gondii* vaccine immunization to intermediate hosts (NHPs, marsupials) in zoos. (4) Feline feces in zoos should be inactivated by burning or high-temperature treatment. (5) Recommend that customs include *T. gondii* infection as a mandatory entry quarantine test for imported animals.

Q4: Figures & Tables: Figure 1 (IHC images) should include scale bars and antibody information.

**Response:** Thanks! Figure 1 now includes scale bars and “Bar = 50 μm” (in Figure 1 legend). The antibody information, the protocol of IHC and some specific details were added in the Methods section (Page 6, Line 20-22).

primary antibody: polyclonal rabbit anti-T. gondii antibody

secondary antibody: anti-rabbit IgG conjugated with HRP/DAB

Q5: Figure 3 (Survival curves) needs log-rank test results.

**Response:** Thanks for your suggestionS. Figure 3 (Survival curves) explanation includes the log-rank test result.

Q6: Grammar needs revision by a native speaker

**Response:** Thanks for your suggestion. We have tried our best to polish the language in the revised manuscript and hope that the correction will meet with approval.

**Reviewer #3:**

Q1: page 8, lines 15-16: Some monkeys were *T. gondii* seronegative, although they died of toxoplasmosis [13]. IHC and PCR may be more sensitive than serological techniques.” You have to discuss this results clearly and support your comment with references.

**Response:** Thanks for your kind comments! The humoral immune response requires at least one to two weeks, and many New World monkeys have not yet produced detectable antibodies when they die of acute toxoplasmosis. We have expanded the Discussion about this point (Page 14, Line 15-22 ).

**Reviewer #4:**

The authors did not report any limitations.
The conclusions are supported by the data presented public health relevance is addressed?

**Response:** The limitations and public health suggestions of this study were added. Please see Page 17, Line 21- Page 18, Line 2.

“The present study on the presence of *T. gondii* in captive NHPs has several limitations. First, there was a limited number of serum and fresh tissue samples for in-depth study. A second limitation is that our understanding of the specificity and sensitivity of the diagnosis (MAT, PCR, mice biology) of *T. gondii* infection in various species of NHPs remains limited. This study may provide a basis for research on *T. gondii* infection in NHPs and humans.”

**Reviewer #5:** (No Response)

**Summary and General Comments**:

**Reviewer #1:**

The authors report toxoplasmosis in non- human in captive primates in China. They report isolation of viable Toxoplasma from tissues of a Black mangabey for the first time from this host; which is of scientific interest. This is detailed investigation using proper methods. Results are worthy of publication, however, the paper is too long. Suggestions for shortening the paper are:
**Response:** Thank you for your suggestions. Regarding manuscript length, we faced a delicate balance: while initially aiming for brevity, other reviewers suggested method details (e.g., IHC, PCR, MAT, Tox isolation, RFLP-PCR). Although we streamlined redundant descriptions, the added more in-depth discussions and description of methods and results ultimately increased the total length.

The methods were mature and have been used for many years in our lab. Corresponding Author (Yurong Yang) had worked in Dr. Dubey Lab (Beltsville, MD, Agricultural Research Service, USDA, USA) during 2013.9-20014.9. Dr. Dubey and Yurong Yang also have co-published papers (n=9, 2014-2025). Yurong Yang Lab masters all these techniques. We acknowledge the challenge in balancing conciseness and have shown more detailed methods.

Q1: Title-First isolation of viable Toxoplasma gondii from a –

**Response:** Thank you for your suggestions! It was revised.

“First Isolation of Viable *Toxoplasma gondii* from a Black Mangabey (*Lophocebus aterrimus*) Reveals the Emergence of the *Africa* 1 Lineage in East Asia”

Q2: page 4, line 4-replace analysis with evaluation

**Response:** It was revised. Thanks! Line 5, Page 18.

Q3: page 4 line 13 replace checked with examined—also in rest of the paper

**Response:** Thank you for your suggestions! We have changed all of “checked” to “examined” in revised text and marked it in blue font.

Q4: Page 5, line 8, replace into with on to

**Response:** We've corrected it in the revised text. Please see Page8, Line12.

Q5: Move Table 1 to Supplemental Table

**Response:** Thank you for your suggestion. We have revised it to Table S1.

Q6: Describe Case 35 in the text

**Response:** As recommended, we have added more information about case#35 in section 3.1, including clinical signs, pathological findings, possible death causes, and limitations. Page 13, Line 12-21.

Q7: Page 7-lines 15-19-replace with a short sentence-findings were confirmed ultrastructurally

**Response:** They were revised and marked blue color in section 3.3. Page12, Line2.

Q8: Figures 1 and 2 move to supplemental Figures

**Response:** Thank you for your suggestions. After detailed consideration, we want retain Figures 1 and 2 in the main text. These figures — showing IHC evidence of T. gondii infection in NHPs and the isolated strain's morphological features.

Q9: Supplemental Table 1-This Table is confusing-Column 1 state if it is based on counted tachyzoites or 10-fold dilutions-remove % ages-it is obvious. Column 2-No. infected/ No. Inoculated. Column 3—remove DPI. Delete last column-state in foot note.

**Response:** Thank you for your suggestions. They were revised and marked with blue color in Table S3.

Q10: Reduce discussion-focus on new findings

**Response:** It was revised.

(1) First isolation of ToxoDB #6 (TgMonkeyCHn3) in East Asia.

(2) New intermediate host: The black mangabey (*Lophocebus aterrimus*) was identified as a new host for *T. gondii*.

(3) Virulence: The survival time of 39.1% (27/69, CI: 28.47%-50.94%) ToxoDB #6 isolates showed no significant differences with TgMonkeyCHn3.

(4) Epidemiological insights: We proposed potential transcontinental transmission through international trade and emphasize loopholes in animal quarantine.

(5) Practical significance: Specific measures (e.g., feral cat control, oocyst inactivation, inspections) are recommended for zoos.

**Reviewer #2:**

The article submitted by Ma et al. is in the field of journal and investigating the *T. gondii* infection in Non-human Primates from Zoo. The authors did serological, molecular and bioassay-based evidence confirming that Black Mangabey (Lophocebus aterrimus) can serve as an intermediate host for *T. gondii*, and they successfully isolate a viable strain (TgMonkeyCHn3). The study highlights the strain’s high virulence and its ToxoDB genotype #6, previously reported in Africa but not in China. While the study presents important findings, there are major concerns regarding novelty justification, statistical analysis, methodology transparency, and interpretation of virulence data that need to be addressed before publication.

**Response:** Many thanks! They were revised and polished.

(1) Novelty: It is the first isolation of ToxoDB #6 in East Asia and the identification of Black Mangabey as a new intermediate host, which expanded this genotype’s known geographic distribution and revealed potential epidemiological origin of transcontinental transmission and emphasized the significance of the emergence of this highly virulent isolate in China.

(2) Statistical analysis: 2.9 Statistical analysis section now includes log-rank (Mantel-Cox) test, Chi-square test. Statistical significance test was added to the Figures, Results and Discussion. 95% confidence intervals for infection rates are now included in the Results.

(3) Methodology transparency: Additional details (e.g., protocols of PCR, IHC, bioassay, in vitro culture, TEM, virulence evaluation and more references) were added to the Methods section.

(4) Virulence interpretation: We have compared the virulence evaluation to other ToxoDB #6 isolates.

Thank you for your valuable guidance.

Q1: The introduction briefly mentions that New World monkeys are more susceptible than Old World monkeys, but does not explore why susceptibility differs (e.g., immune system variations, parasite strain differences). If it is something to do with the parasite strain, there should be more reference about this issue.

**Response:** Thank you for your suggestion. In the revised manuscript, It was added on host-parasite co-evolution as a explore of susceptibility differences between New World and Old World NHPs. Please see Page4, Line14-19.

Q2: Authors states that this is the first report of genotype #6 in China, but does not compare findings with previous *T. gondii* genotyping studies in China or NHP studies worldwide. If there is any article mentioned on genotype#6, especially in China?

**Response:** Thank you for your comments. It was revised, Page4, Line21-23, Page5, Line1-4.

The summary of *T. gondii* genotyping in China was published in previously paper: Yang L, et al. Isolation and Genetic Characterization of *Toxoplasma gondii* from a Patas Monkey (*Erythrocebus patas*) in China. Genes (Basel). 2023, 14(8):1606.

[**https://www.mdpi.com/article/10.3390/genes14081606/s1**](https://www.mdpi.com/article/10.3390/genes14081606/s1),

Table S1: Summary of viable *T. gondii* isolates (n = 169) from animals and humans in China

Among *T. gondii* isolates (n = 169) from animals and humans in China, at least more than 16 ToxoDB RFLP-genotypes were found. These include ToxoDB RFLP-genotypes #1–#5, #9, #10, #17–#18, #20, #204–#205, #292, #319, and mix genotypes, and no prior studies in China have reported ToxoDB #6.

**Reviewer #3:**

This is an interesting *T. gondii* isolation study from non-humans primates. There are some issues that should be clarified. My comments are attached.

The Title “Direct evidence of Black mangabey (Lophocebus aterrimus) as intermediate host of Toxoplasma gondii through isolation viable strain” should be changed as “Demonstration of Black mangabey (Lophocebus aterrimus) as an intermediate host of Toxoplasma gondii through live strain isolation”

**Response:** Thank you for your suggestions! It was revised to:

“First Isolation of Viable *Toxoplasma gondii* from a Black Mangabey (*Lophocebus aterrimus*) Reveals the Emergence of the *Africa* 1 Lineage in East Asia”

We added "Emergence of Africa 1 lineage" because it explicitly connects the isolated strain to its potential epidemiological origin (from Africa), which aligns with the host's native range, highlighting the likely transcontinental transmission through wildlife trade and the public health challenge of this virulent isolate. We believe that this new title reflects the main findings and highlights of the article.

**Reviewer #4:**

This study is important in terms of the transmission dynamics of *T. gondii*. It also contains important results in terms of public health, as it emphasizes that there may be new hosts in natural life and zoos and that they may differ in terms of virulence. However, further detailing the method section and explaining the workflow with a picture would improve the quality of the article.

**Response:** Thank you for your suggestions. According to your nice suggestions, detailed protocols are now provided in the Methods section, including PCR, IHC, MAT, bioassay, in vitro cultivation, genotyping, TEM, virulence evaluation in mice and statistical analysis. In addition, we have added a flowchart (Fig S1).

**Reviewer #5:**

The authors conducted a very comprehensive study to demonstrate the presence of *T. gondii* in NHM and to isolate and genotype *T. gondii*. According to the results, the presence of *T. gondii* in NHM was detected by different diagnostic methods and ToxoDB genotype #6 was identified. I think the manuscript can be accepted for publication, but major revision are needed. The recommendations are as follows.

Q1: The introduction is very weak and should be extended. *T. gondii* genotypes detected in NHP in previous studies should be mentioned.

**Response:** Thank you for your positive comments and valuable suggestions! We have included more previous studies in the introduction, including the genotypes of the isolates from NHPs reported to date.

Q2: Please check the statement “75.0-98.5% genetic homology”.

**Response:** Thanks! It was revised.

“NHPs serve as a critical translational bridge between fundamental research and human clinical applications, owing to their phylogenetic proximity and high degree of genetic homology with humans.”

Q3: Tissue homogenization for bioassay should be specified in more detail.

**Response:** Thank you for your comments! As recommended, we have added more detailed protocol about bioassay in section 2.5. The revised methods are as follows:

“Tissue samples (heart, tongue, brain, and leg muscle) from five NHPs were bioassayed in mice. Briefly, the tissue homogenate (50 g) was digested with pepsin (5.2 g hog stomach pepsin, 10.0 g NaCl, and 14 mL HCl, with water added to a volume of 1000 mL, pH 1.10–1.20) at 37°C for 60 min with shaking [1]. Subsequent filtration through gauze and centrifugation separated the sediment, which was neutralized using sodium bicarbonate (1.2%, pH 8.3) with a phenol red indicator (producing a color change to orange). IFN-γ knockout mice were used for bioassay, as they lacked Th1 immune responses, leading to parasite replication and large numbers of tachyzoites for *in vitro* culture [1]. The pepsin-digested products (1 mL per mouse) were subcutaneously injected into *T. gondii-*free Swiss mice (n = 4–5) and/or IFN-γ knockout mice (n = 1–2).”

Q4: The genotyping methodology should be specified in more detail.

**Response:** Thank you for your suggestions. They were revised and added.

Q5: What is ToxoDB genotype #6? Is it genotype I, II, III or atypical genotype? Please provide more detailed information.

**Response:** Thank you for your comment. ToxoDB genotype #6 belongs to Type I variant, Clade A (Ihara et al., 2024).

Based on the traditional identification, ToxoDB genotype #6 belongs to atypical genotype, but strictly speaking, it is a genotype of the ToxoDB naming system. The traditional genotyping for *T. gondii* classifies strains into three major clonal lineages (Type I, II, and III) using a limited set of genetic markers. It was widely used in early studies but had major limitations for reliable strain characterization, particularly in regions like South America and Asia, where atypical or recombinant strains are common.

In contrast, the ToxoDB classification (http://www.toxodb.org) employs a high-resolution, multilocus PCR-RFLP approach using 10 genetic markers, enabling the identification of over 300 genotypes globally. For strains defined by ToxoDB RFLP, a specific numeral is attributed to each different genotype according to the codification adopted by the ToxoDB naming system. The ToxoDB method’s strength lies in its ability to resolve atypical or recombinant strains and trace zoonotic transmission pathways.

Ihara F, Kyan H, Takashima Y, Ono F, Hayashi K, Matsuo T, Igarashi M, Nishikawa Y, Hikosaka K, Sakamoto H, Nakamura S, Motooka D, Yamauchi K, Ichikawa-Seki M, Fukumoto S, Sasaki M, Ikadai H, Kusakisako K, Ohari Y, Yoshida A, Sasai M, Grigg ME, Yamamoto M. Far-East Asian Toxoplasma isolates share ancestry with North and South/Central American recombinant lineages. Nat Commun. 2024 May 22;15(1):4278.

Q6: It is not clear what Tox# 20-53 or Tox# 20-54 are. Please correct such statements.

**Response:** Thank you for your suggestions. For ease of understanding, the naming logic was explained in the Methods section 2.5 (Page 7, Line 21-Page 8, Line 1): “Each group was sequentially named Tox#20-x. For example, in “Tox#20-50”, “Tox” represents *T. gondii* bioassay; “20” represents the species ID of the NHPs, and “50” represents the group ID.”

Once again, we deeply appreciate the reviewers’ opinions and time invested in evaluating our work. With your enthusiastic and professional guidance, the MS was improved and polished. We sincerely look forward to your responses.

Yours sincerely,

Yiheng Ma, Liulu Yang, Yurong Yang

Laboratory of Veterinary Pathology

College of Veterinary Science

Henan Agricultural University

Zhengzhou

China

450002
