# Supplementary material for: First isolation of viable Toxoplasma gondii from a black mangabey (Lophocebus aterrimus) reveals the emergence of the Africa 1 lineage in East Asia
Source: PLoS Negl Trop Dis. 2025 Jul 23;19(7):e0013133. doi: 10.1371/journal.pntd.0013133 (PMC12286360; doi:10.1371/journal.pntd.0013133)
Supplement: Attachment — Submitted filename: 3 Response to Reviewers.docx [file pntd.0013133.s013.docx]

PNTD-D-25-00020

Dear Editors and Reviewers,

We have attempted to clarify all the questions in the text and colored the revision part (Blue color). We hope to have clarified all the questions. Thank you for your kind help.

**Methods, Results, Conclusions:**
**Reviewer #1, #2,#3，#4, #5**: ok

**Response:** Thank you! We sincerely appreciate the reviewers' comments and positive suggestions. These opinions encourages us to do more research about the topic of *Toxoplasma gondii* and wild animals health.

**Editorial and Data Presentation Modifications:**
Reviewer #1: 1. Remove all duplication
2. Figures 1 and 2 move to supplementary section
3 Condense mouse mortality discussion
Swiss Webster outbred mice

**Response:** They were revised and marked with blue color.

Figures 1 and 2 were moved to supplementary section.

The MS was polished and concised.

**Reviewer #2,#3，#4, #5**: ok

**Response:** Thank you!

**Summary and General Comments:**
**Reviewer #1,#2,#4, #5**: ok

**Response:** Thank you!

**Reviewer #3:**There are publications where Africa 1 strain have been isolated from humans and stray cat which is outside Africa. You have not discussed these results in your discussion section.

**Response:** Thanks for your kindly suggestions! It was added and revised, the information from Turkey.

**Figure resubmission:**

**Response:** They were checked and downed from Web.

https://pacev2.apexcovantage.com/.

Yours sincerely,

Yurong Yang

Laboratory of Veterinary Pathology

College of Veterinary Science

Henan Agricultural University

Zhengzhou

China

450002
